# Enhanced optical gradient forces between coupled graphene sheets

**DOI:** 10.1038/srep28568

**Published:** 2016-06-24

**Authors:** Xinbiao Xu, Lei Shi, Yang Liu, Zheqi Wang, Xinliang Zhang

**Affiliations:** 1 Wuhan National Laboratory for Optoelectronics, Huazhong University of Science and Technology, Wuhan, 430074 China

## Abstract

Optical gradient forces between monolayer infinite-width graphene sheets as well as single-mode graphene nanoribbon pairs of graphene surface plasmons (GSPs) at mid-infrared frequencies were theoretically investigated. Although owing to the strongly enhanced optical field, the normalized optical force, *f*_*n*_, can reach 50 nN/μm/mW, which is the largest *f*_*n*_ as we know, the propagation loss is also large. But we found that by changing the chemical potential of graphene, *f*_*n*_ and the optical propagation loss can be balanced. The total optical force acted on the nanoribbon waveguides can thus enhance more than 1 order of magnitude than that in metallic surface plasmons (MSPs) waveguides with the same length and the loss can be lower. Owing to the enhanced optical force and the significant *n*_*eff*_ tuning by varying the chemical potential of graphene, we also propose an ultra-compact phase shifter.

When light propagates in parallel waveguides, the waveguides can feel the optical gradient force. The force is generated by the fact that a dipole in an inhomogeneous electric field will experience a force in the direction of the field gradient^1^. The force can be attractive force or repulsive force depending on whether the relative phase difference of corresponding guided modes in two waveguides is 0 or π^2^. It provides us with a new dimension to manipulate light in photonic integrated circuits^3^,^4^,^5^,^6^,^7^. To manipulate light obviously, large optical force is desired^8^,^9^. It has been demonstrated that the optical force can be enhanced by use of slow light^10^, cavity resonance^11^, metamaterials^12^ and surface plasmon polaritons (SPPs)^13^,^14^. Due to the deep optical energy confinement of metallic surface plasmons (MSPs), the optical force can be enhanced about one order of magnitude. As we know, graphene can also support graphene surface plasmons (GSPs) at infrared frequencies^15^,^16^,^17^,^18^.

Graphene, a single layer of carbon atoms with honeycomb lattice, is a fascinating material and has attracted considerable attention as its exceptional electric and photonic properties, such as ultrahigh electron mobility^19^, strong optical nonlinearity^20^, and high thermal conductivity^21^. As we know, the parameters of traditional metals are hardly tunable. However, the optical response of graphene is depending on carriers density, so the most remarkable advantage of graphene over metals is the ability to tune the conductivity dynamically by gate voltage, chemical doping, electric field and magnetic field^22^, etc. Compared with the SPP waves in noble metals, when graphene’s carrier density is elevated (i.e., the Fermi energy or chemical potential gets larger), the propagation losses of GSPs can be lower than those of MSPs^18^. In addition, plasmons propagate in graphene with the speed comparable to Fermi velocity (*v*_*F*_ = 9.5 × 10^5^ m/s)^23^, which is much smaller than the speed of light in vacuum, this makes the volumes of plasmons in graphene are several orders of magnitude smaller than those in noble metals. In consequence of such good properties, graphene can be used in electro-optic modulator^24^, transformation optics^25^, broadband polarizer^26^, optical nano-imaging^27^,^28^, plasmon-induced transparency^29^,^30^, etc. Besides, there are many significant theoretical researches about graphene sheet arrays and coupled graphene pairs come to light^23^,^31^,^32^,^33^, and some interesting phenomena such as the strong coupling effect^34^, plasmon-negative refraction effect^35^ were found.

However, the characterization of the optical gradient force between coupled graphene sheets has not been reported. In this paper, we study the optical gradient force between coupled graphene sheets theoretically and numerically. Firstly, we investigate the optical force of GSPs mode between infinite-width single-layer graphene sheets. Besides, the optical force between single-mode graphene nanoribbon waveguide are also studied. Finally, we propose an ultra-compact phase shifter.

## Results and Discussion

In this paper, we choose the temperature *T* = 300 K, the electron mobility *μ* = 30000 cm^2^/s^−1^V^−1^, which is relative conservative, for *μ*>100000 cm^2^/s^−1^V^−1^ has been experimentally achieved^19^. Besides, unless otherwise stated, we set the chemical potential *μ*_*c*_ = 0.15 eV corresponding to relaxation time *τ* = 0.5 ps and the thickness of graphene is *∆* = 0.5 nm in the simulation.

### Optical force between infinite-width monolayer graphene pairs

**Figure 1:**
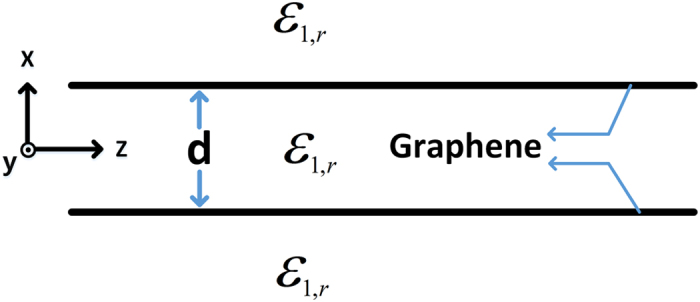
The schematic of coupled infinite-width monolayer graphene sheets. The relative permittivity of the surroundings is *ε*_*1,r*_. The gap between graphene sheets is *d*. Light propagates along the *z* direction.

According to the optical waveguide theory, graphene is treated as a thin surface layer characterized by a surface conductivity σ_g_(ω) (see Methods). As is shown in Fig. 1, we get the dispersion relation of both the TE and TM mode GSPs by solving the Maxwell equations with corresponding boundary conditions (see Supplementary Information):


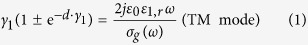



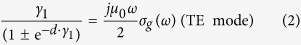


where 
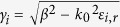
 and “±” corresponding to anti-symmetric mode and symmetric mode^2^.

No matter from the quantum “photon” picture or the purely classical picture, two derivation methods give the same result that for a closed system of two waveguides separated by distance *d,* the energy conservation law implies that the optical gradient force on either waveguide is given by^2^,^11^,^36^:


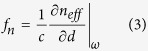


*f*_n_ (N/m/W) is the optical force per unit length normalized to the local power. When the propagation loss is small, the local power *P* at any point along the waveguide length *L* can be treated as a constant. It is convenient to use *f*_n_ to estimate the total optical force *F*_*n*_ acting on waveguides by *F*_n_ = *f*_n_ · *L* · *P*.

If the waveguide is lossy, from equation (3) we can find that although the *f*_*n*_ is still a constant along the waveguide, but the power of light will decay exponentially while propagating: *P*(*L*) = *P*(0)*e*^−*αL*^. Obviously, loss will affect the total optical force acted on the waveguide. The propagation length were defined by: *L*_m_ = *α*^−1^ = 1/(2|Im(*β*)**|**)^23^, at where the power is *e*^−1^ of the initial power.

Besides, by using the classical electrodynamics to derive the conservation law for linear momentum in an optical field, the optical force can also be calculated based on the finite-element method (FEM), i.e. By integrating the Maxwell’s stress tensor (MST) 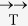 around arbitrary surface enclosing the waveguide COMSOL^1^:


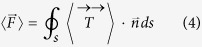



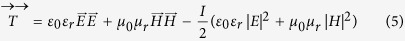


where 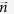 is the outward unit normal to the surface. The MST method is general, so it is also suitable for graphene.

**Figure 2:**
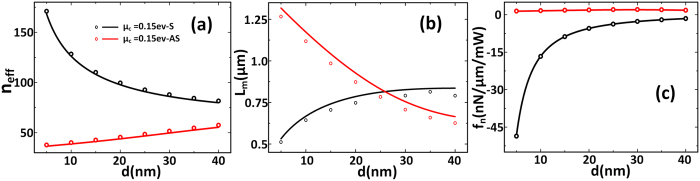
The results of TM mode GSPs of coupled infinite-width monolayer graphene versus different waveguide gap. The solid lines are theoretical calculation results calculated by equation (1) and equation (3). The open symbols are simulation results calculated by COMSOL where the optical force is calculated by MST method. (Black: symmetric mode, Red: anti-symmetric mode). (**a**) The effective refractive index of GSPs. (**b**) The propagation length of GSPs. (**c**) The normalized optical gradient force versus the gap of graphene pairs.

For TM mode GSPs. We consider the wavelength *λ*_*0*_ = 10 μm^32^,^34^. The results are shown in Fig. 2, the normalized optical force calculated by equation (1) and equation (3) matches the simulation results by MST method very well. *n*_*eff*_ is the effective refractive index of anti-symmetric mode or symmetric mode. As is shown in Fig. 2(a), *n*_*eff*_ is 2 orders of magnitude larger than that of conventional silicon waveguides, so the wavelength of the guided plasmonic mode *λ*_*GSPs*_ = *λ*_*0*_/*n*_*eff*_ is much smaller. This means the confinement of light is very strong and can also lead to a larger propagation loss and consequently shorter propagation length, especially for the symmetric mode. So there is a trade-off between *n*_*eff*_ and the *L*_*m*_ according to Fig. 2(a,b).

From Fig. 2(a) we can see that the slope of the symmetric mode is larger than that of the anti-symmetric mode, this means the optical field gradient of the former is larger. So it is shown in Fig. 2(c) that the normalized optical force of symmetric TM mode GSPs, *f*_*n.s*_, trending to attract graphene pairs together is stronger than that of anti-symmetric GSPs mode *f*_*n.as*_, and *f*_*n.s*_ is more sensitive to the gap compared to *f*_*n.as*_. As the evanescent field gradient decays with the increased distance away from the waveguide, the optical force also decays when the gap increased^2^.The maximum of *f*_n_ is about −50 nN/μm/mW, which is about 1 order of magnitude larger than the optical force between coupled metallic waveguides^37^. To our best knowledge, this is almost the biggest normalized optical force among have been reported.

**Figure 3:**
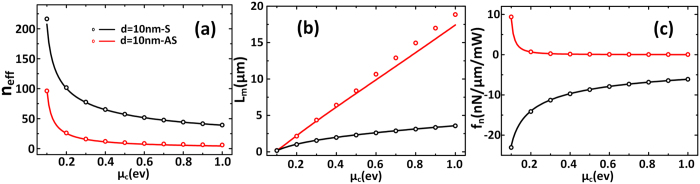
The results of TM mode GSPs of coupled infinite-width monolayer graphene versus different chemical potential. The solid lines are theoretical calculation results calculated by equation (1) and equation (3). The open symbols are simulation results calculated by COMSOL where the optical force is calculated by MST method. (Black: symmetric mode, Red: anti-symmetric mode). (**a,c**) The effective refractive index, propagation length and the normalized optical gradient force versus chemical potential with *d* = 10 nm, respectively.

However, we can see from the Fig (2) that although the normalized optical force is large, but it brings large propagation loss too. As we know, the most remarkable advantage of graphene is the capability to tune its chemical potential, i.e., carrier density. In Fig. 3(a,c) we analyze the influences of different chemical potentials on the optical and mechanical performances. For two graphene sheets separated by 10 nm, when chemical potential increases from 0.1 ev to 1 ev, *n*_*eff*_ decreases and the propagation length increases obviously from 0.15 μm to 19 μm at most. Meanwhile, the normalized optical force is decreased. So the optical force and the optical loss can be controlled by changing the chemical potential. This gives us a new way to control the optical force.

Besides, a weakly guided TE mode GSPs with very low propagation loss can exist in graphene^26^,^38^,^39^. By contrast, only TM modes can be supported in metals. As previous work proved that the confinement of TE mode GSPs is so weak that it propagates along the 2D graphene layer with the speed close to the velocity of light^38^.This means *n*_*eff*_ ≈ 1. By equation (2), When *λ*_*0*_ = 4.5 μm (*σ*_*g,i*_ < 0) the effective refractive index is about 1.0046, which is very close to the refractive index of air. The GSPs modes are almost cut off. So most of the energy is in the air around the graphene pairs and there is almost no optical field gradient around graphene. Hence, the result is understandable that the normalized optical force *f*_*n*,*s*_ ≈ −8 × 10^−10^ nN/μm/mW is about 10 orders of magnitudes smaller than that of TM mode GSPs, and thus the optical force of TE mode GSPs can almost be ignored.

### Optical force between graphene nanoribbon pairs

**Figure 4:**
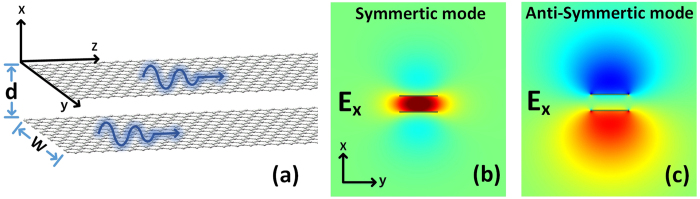
The schematic of coupled graphene nanoribbon sheets and the electric field x component of symmetric mode and anti-symmetric mode. (**a**) The schematic of the top-bottom configuration with width *W* and gap *d*. (**b,c**) The electric field *x* component of symmetric mode and anti-symmetric mode, respectively.

In the following, we consider the top-bottom configuration with limited width graphene as is shown in Fig. 4(a). Graphene nanoribbons can support both waveguide GSPs (WGSPs) modes and strongly localized edge GSPs (EGSPs) modes^40^. When the width of graphene nanoribbon *W* is smaller than 50 nm, the ribbon become a single-mode waveguide^32^ that only EGSPs mode can be supported. *d* is the gap between two graphene nanoribbons. In our analysis we consider the single-mode graphene nanoribbon without referring to the type of the edge (e.g., zigzag edge or armchair edge) and the optics wavelength is *λ*_*0*_ = 10 μm. All the following results are simulation results calculated by COMSOL and the optical force is calculated by the MST method of equation (4).

**Figure 5:**
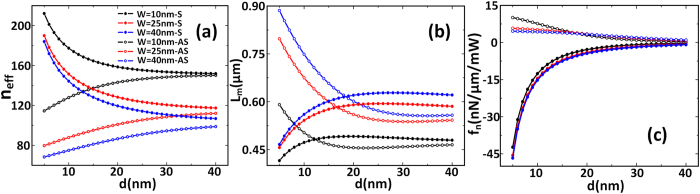
The results of coupled single mode graphene nanoribbons with different width versus gap. (**a**–**c**) The effective refractive index, propagation length and the optical gradient force density versus the waveguide gap with *μ*_*c*_ = 0.15 *e*V, respectively. Solid symbols: symmetric mode. Open symbols: anti-symmetric mode.

As the optical force is related to the geometry of nanoribbon. From Fig. 5(a,b) we find that for a specific *d*, *n*_eff_ decreases with increasing *W*, which is different to silicon waveguide. For the nanoribbon with a specific *W*, the effective refractive index converges to a certain value quickly as the gap increases, this means the optical field gradient is very large.

In Fig. 5(c), due to the stronger field confinement and larger field gradient, *f*_*n*_ between graphene nanoribbon pairs is about 20 times of that between coupled rectangular plasmonic waveguides^37^, 2 orders of magnitude of that between the coupled hybrid plasmonic waveguides^14^ and about 3 orders of magnitude of that between the coupled silicon waveguides^2^,^37^, the maximum *f*_n_ can reach −45 nN/μm/mW. However, just like in infinite-width single-layer graphene pairs, when *μ*_*c*_ = 0.15 *e*V, the propagation length is still very short as is shown in Fig. 5(b).

**Figure 6:**
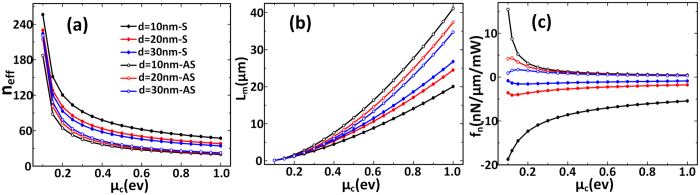
The results of coupled single mode graphene nanoribbons with different waveguide gap versus chemical potential. (**a**–**c**) The effective refractive index, propagation length and the normalized optical gradient force versus chemical potential with *W* = 25 nm, respectively. Solid symbols: symmetric mode. Open symbols: anti-symmetric mode. By changing the chemical potential we can balance the normalized optical force and the optical loss.

Similarly to the infinite-width graphene sheets, we analyze the influences of different chemical potentials on the optical and mechanical performances again. With the chemical potential increases from 0.1 ev to 1 ev, the variation tendency of the results in Fig. 6(a,c) is analogous to that in Fig. 3(a,c) that n_eff_ decreases rapidly and the propagation length increases. Although the normalized optical force is decreased, but it is still on the order of nN/μm/mW and the propagation length increases obviously from 0.15 μm to 41 μm at most.

As is shown in Fig. 6(b,c) that small chemical potential brings large *f*_n_, but it brings large propagation loss at the same time, So *f*_n_ cannot be used to evaluate the magnitude of the total optical force *F*_*n*_. In fact, the total optical force acting on the nanoribbon waveguides per unit input optical power, *F*_*n*_, is calculated as follow:


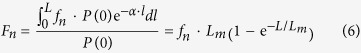


Equation (6) shows that *F*_*n*_ is related to *f*_*n*_, *L*_*m*_ and the propagation distance *L*. This means the normalized optical force and the optical loss can be balanced by changing the chemical potential to get a larger total optical force.

**Figure 7:**
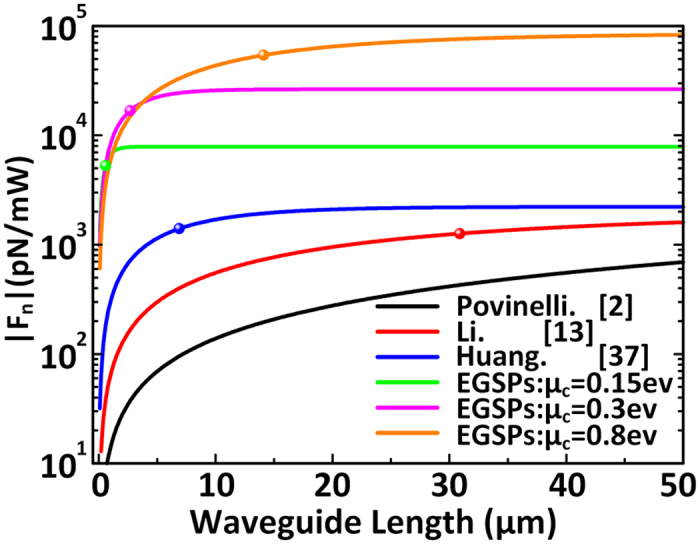
The compare of total optical force acting on the waveguides versus the waveguide length in different waveguide structures. The horizontal ordinate value of colorful balls is as large as the *L*_*m*_ of each structure.

In Fig. 7 the comparison of *F*_*n*_ versus waveguide length in different structures that have been reported is shown. All the waveguide gap is 10 nm. The black curve, red curve and blue curve represents all-dielectric waveguides, metal-dielectric hybrid plasmonic waveguides and all-metallic SPP waveguides, respectively. The other three curves are graphene nanoribbon waveguides with different chemical potentials. The horizontal ordinate value of colorful balls on the curves is as large as the *L*_*m*_ of each structure.

As is shown in Fig. 7, for coupled waveguides about 50 μm, the maximum *F*_n_ between graphene nanoribbon can enhance at least 1 order of magnitude compared with that between coupled metal waveguides, 2 orders of magnitude compared with that between coupled silicon waveguides, and we believe larger *F*_n_ can be achieved for larger *μ*_c_.

**Figure 8:**
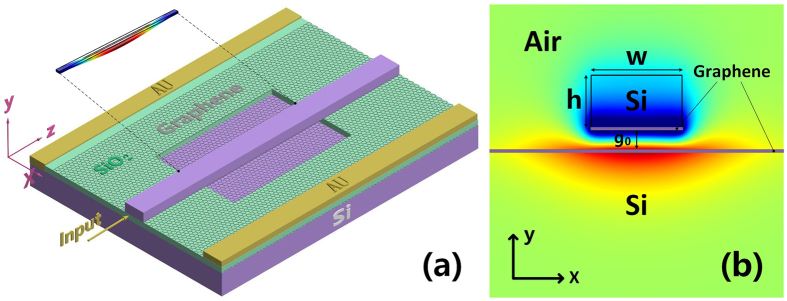
The optomechanical phase shifter. (**a**) The three dimensional schematic illustration of the device, the inserted figure is the deflection of the double-clamped beam calculated by COMSOL. (**b**) The mode profile in cross section of the free-standing part at wavelength *λ*_*0*_ = 10 μm.

Owing to the large optical force between graphene sheets and the significant *n*_*eff*_ tuning by varying the chemical potential of graphene, we propose an ultra-compact phase shifter working in mid-infrared spectral region. Figure 8(a) is the three dimensional schematic illustration of the device, the inserted figure is the deflection of the double-clamped beam calculated by COMSOL. The phase shifter can be fabricated with a series of standard semiconductor fabrication processes. The graphene sheets can be grown by the chemical vapor deposition (CVD) method and mechanically transferred onto the substrate. The residual graphene can be removed by oxygen plasma. The stack of graphene and SiO_2_\Si can be deposited by the plasma-enhanced chemical vapor deposition (PECVD) and electrodes can also be positioned to connect with graphene. By etching the SiO_2_ substrate with hydrofluoric (HF) acid the free-standing double-clamped beam will be obtained^3^.

Figure 8(b) is the mode profile in cross section of the free-standing part at wavelength *λ*_*0*_ = 10 μm. There are two layers of graphene separated by a SiO_2_ layer. The upper layer graphene on the groove region excluding the part below the free-standing beam is removed before HF acid etching. So a graphene nanoribbon adsorbed under the Si waveguide by van der Waals force (VWF) is obtained. Besides, although the lower layer graphene is broad, it becomes a soft-boundary graphene nanoribbon^41^,^42^ as the existence of the free-standing Si waveguide. Once the plasmonic waves between two graphene nanoribbons are excited, the device will work due to the optical gradient force^43^ and the voltage turning.

**Figure 9:**
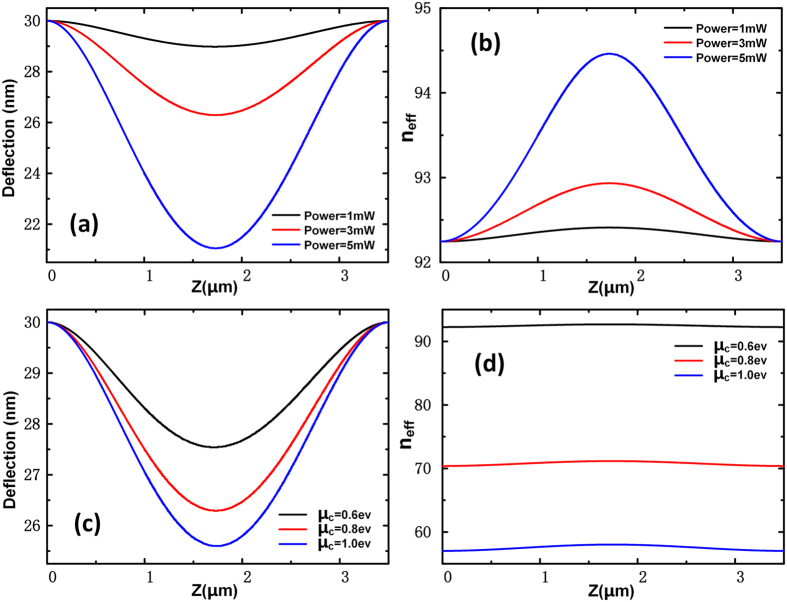
The performance of optomechanical phase shifter. (**a**) Deflection curve of the phase shifter in the cases of *μ*_*c*_ =  0.8 *e*V and different incidence power. (**b**) The effective refractive index along the free-standing waveguide in the case of (**a**). (**c**) Deflection curve of the phase shifter in the cases of incidence power 3 mW and different *μ*_*c*_. (**d**) The effective refractive index along the free-standing waveguide in the case of (**c**).

In our analysis, the width of the free-standing Si waveguide is 100nm and the height is 50 nm. As the strong optical field confinement, the initial separation between two graphene layers g_0_ is 30 nm. Because of the large propagation loss of GSPs, we set the length of the free-standing waveguide *L* = 3.5 μm. The light wavelength *λ*_*0*_ = 10 μm. Figure 9(a) shows the deflection curves of the double-clamped beam calculated by COMSOL with the *μ*_*c*_ = 0.8 *e*V and different incidence powers. As the *n*_eff_ of the coupled graphene nanoribbons vary with the distance between the graphene sheets. Figure 9(b) is the corresponding effective refractive index along the waveguide under these conditions. We can see that although the deflection of the beam is small, it brings very large effective refractive index variation. The *n*_eff_ variation in conventional Si waveguide with the same deflection is about 0.01 when light wavelength *λ* = 1.55 μm. Besides, if we change the chemical potential of graphene, the variation of effective refractive index can be larger. In Fig. 9(c,d), we analyze the performance of the device with different chemical potential when incidence power is 3 mW. In Fig. 9(d), we found that without the consideration of beam deflection caused by optical force, changing *μ*_*c*_ from 0.6 *e*V to 0.8 *e*V makes the *n*_eff_ vary from 57 to 70. Although different *μ*_*c*_ makes different optical forces as shown in Fig. 9(c), but the *n*_eff_ variation caused by different optical force is much smaller than that caused by different *μ*_*c*_. We can finally obtain the phase shift due to different *μ*_*c*_ or power with the following equation:


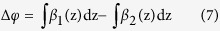


where Δ*φ* is equal to *k*_*0*_∙*S*, *S* is the area between two curves with propagation constants *β*_*1*_*(z)* and *β*_*2*_*(z)*.

After calculation by Equation (7), we found that in Fig. 9(b) when incidence power changed from 1 mW to 3 mW, the phase shift was Δφ_3mW,1mW_ = 0.2π and Δφ_5mW,1mW_ = 0.7π. In Fig. 9(d) when *μ*_*c*_ changed from 0.6 *e*V to 0.8 *e*V, the phase shift was Δφ_0.8*e*V,0.6*e*V_ = 15.1π and Δφ_1*e*V,0.*6e*V_ = 22.4π.

So we can realize a large-range phase tuning by changing chemical potential, and a precise phase tuning by changing optical power. In many interference structures, a *π* phase shift is enough, so both changing optical power and chemical potential are all feasible. The mechanical vibration frequency *ω*_*j*_ = 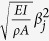 is always less than 1GHz, where *β*_*j*_ obeys the equation: cos(*β*_*j*_*L*)∙cosh(*β*_*j*_*L*) = 1, *E* is the Young’s Modulus, *I* is the cross sectional area moment of inertia with respect to the neutral axis, *ρ* is the density, *A* is the cross-sectional area. But in many situations, we need to realize the phase shifting with high frequency, or the optical power is not convenient to change, then the way of chemical potential tuning is preferable. According to 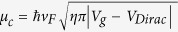, we can control the chemical potential by applied voltage, where *V*_Dirac_ = 0.8 V caused by natural doping, η = 9 × 10^16^ v^−1^m^−2^ 44, *V*_*g*_ would be considered as the applied voltage. By electrically tuning the Fermi level of the graphene sheet, the modulation frequency of the guided light over 1 GHz had been demonstrated^44^. As a result, without resonance structure our device also has a broad working bandwidth. The comparison of the Casimir forces, VWF, optical force and electrostatic force caused by dynamic tuning of chemical potential is shown in Supplementary Information.

## Conclusion

In this paper, we investigate the optical gradient force between single-layer graphene sheets as well as graphene nanoribbon pairs at mid-infrared frequencies. The dispersion relation of coupled single-layer graphene sheets for TM mode GSPs and TE mode GSPs were derived. We found that due to strong field enhancement and large field gradient, *f*_*n*_ for TM mode GSPs and EGSPs between graphene sheets is more than 1 order of magnitude larger than that in previous plasmonic waveguides. However, TE mode GSPs in the graphene sheet is so weak that the mode is almost cut off. The *f*_*n*_ of TE mode GSPs between graphene sheets is 10 orders of magnitudes smaller than that of TM mode GSPs, thus the optical force of TE mode GSPs can be ignored. Besides, by changing the chemical potential, *f*_*n*_ and the optical propagation loss can be balanced and the total optical force acted on the nanoribbon waveguides can therefore be enhanced. Finally, owing to the enhanced optical force between graphene sheets and large *n*_*eff*_ tuning by varying the chemical potential of graphene, we propose an ultra-compact broadband phase shifter, which can work by both optical and electrical tuning.

## Methods

### Graphene conductivity

Graphene conductivity *σ*_*g*_(ω) consists of two parts: *σ*_*inter*_(ω) and *σ*_*intra*_(ω) related to inter-band transition and intra-band transition, respectively, and it can be derived from the Kubo’s formula^22^,^26^. In the condition of *k*_*B*_*T* ≪ |*μ*_*c*_|:


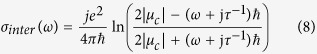



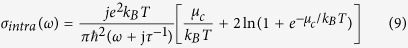


where -*e* is the charge of an electron, *j* is the imaginary unit, *k*_*B*_ is the Boltzmann’s constant, *T* is the temperature, *ℏ* is the reduced Planck’s constant, *ω* is the radian frequency, *τ* = *μμ*_*c*_/*ev*_*F*_^45^ is the relaxation time, which is a function of electron mobility *μ* and the chemical potential or Fermi energy *μ*_*c*_. *Γ* = 1/(2*τ*) is the charged particle scattering rate representing the loss mechanism. The relative dielectric constant of graphene can be derived from the Maxwell’s equations^25^,^34^:


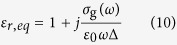


where *ε*_*0*_ is the vacuum dielectric constant, *∆* is the thickness of single-layer graphene. When *λ* = 10 μm, the real part of *ε*_*r,eq*_ is negative, so graphene is metallic and can support TM mode GSPs, when 4.3 < *λ* < 4.95 μm, a weakly guided TE mode GSPs with very low propagation loss can exist in graphene^26^,^38^,^39^.

## Additional Information

**How to cite this article**: Xu, X. *et al*. Enhanced optical gradient forces between coupled graphene sheets. *Sci. Rep.* **6**, 28568; doi: 10.1038/srep28568 (2016).

## Supplementary Material

Supplementary Information
