# Supplementary material for: Enhanced optical gradient forces between coupled graphene sheets
Source: Sci Rep. 2016 Jun 24;6:28568. doi: 10.1038/srep28568 (PMC4919633; doi:10.1038/srep28568)
Supplement: Supplementary Information [file srep28568-s1.pdf]

Supplementary Information

Enhanced optical gradient forces between coupled graphene sheets

Xinbiao Xu, Lei Shi [*], Yang Liu, Zheqi Wang, and Xinliang Zhang

Wuhan National Laboratory for Optoelectronics, Huazhong University of Science and Technology, Wuhan 430074, China

[*]lshi@hust.edu.cn

1. The dispersion relation of GSPs in coupled infinite-width monolayer graphene sheets:

To calculate the optical gradient force theoretically, we should know the dispersion relation of GSPs in coupled infinite-width monolayer graphene sheets. There are two ways to treat the graphene to derive the dispersion equations. In the first way graphene is treated as a traditional waveguide with thickness *T*[1]. By matching the boundary conditions, and figuring out the limit as *T* to zero the dispersion relation could be achieved. The equation can also be derived by supposing that graphene is an interface characterized by the surface conductivity[2,3]. As shown in FigS1.(a), two dielectric waveguides are covered with graphene, and graphene is treated as a thin surface layer characterized by a surface conductivity $\sigma_g(\omega)$. After the dispersion relation of the structure in FigS1.(a) is obtained, we take the limit of $t\rightarrow 0$ to get the dispersion relation of GSPs in coupled infinite-width monolayer graphene sheets as shown in FigS1.(b)[3].

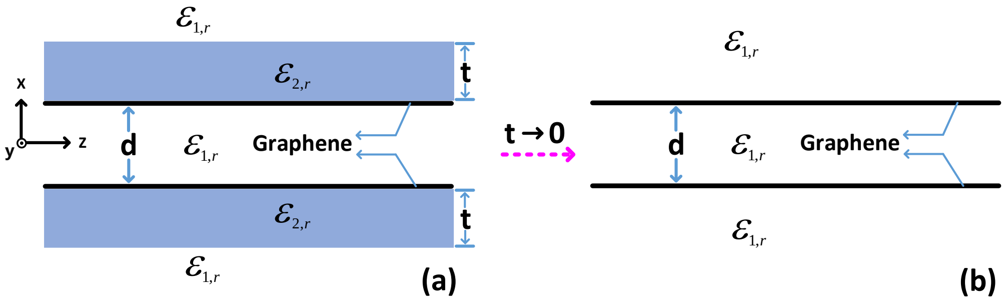

Fig.S1. The schematic that we derive the dispersion relationship of coupled infinite-width monolayer graphene sheets. (a) The schematic of coupled slab waveguides covered with graphene. *t* is the thickness of the dielectric slab. *d* is the waveguide gap. (b) The schematic of coupled infinite-width monolayer graphene sheets. The relative permittivity of the dielectric slabs and the surroundings are $\varepsilon_{2,r}$ and $\varepsilon_{1,r}$, respectively.

The electromagnetic field of the eigenmode has the form of $\vec{\phi}(x)e^{j(\beta z-\omega t)}$ where ϕ(x) is *E*(x) or *H*(x).

Light propagates along the z direction. According to optical waveguide theory, we can get the following electric field distribution of the coupled waveguides. We consider the TE mode first:

$$E_y(x)=\begin{cases} Ae^{-\gamma_1(x-t-d/2)} & t+d/2<x<+\infty \\ Be^{\gamma_2(x-t-d/2)}+Ce^{-\gamma_2(x-d/2)} & d/2<x<t+d/2 \\ De^{\gamma_1(x-d/2)}+Ee^{-\gamma_1(x+d/2)} & -d/2<x<d/2 \\ Fe^{\gamma_2(x+d/2)}+Ge^{-\gamma_2(x+t+d/2)} & -t-d/2<x<-d/2 \\ He^{\gamma_1(x+t+d/2)} & -\infty<x<-t-d/2 \end{cases} \quad \text{(S1)}$$

where $\varepsilon_{i,r}$ is the relative dielectric constant of the material *i*, $\gamma_i=\sqrt{\beta^2-k_0^{\,2}\varepsilon_{i,r}}$.

$$H_z(x)=\frac{-j}{\omega\mu_0}\frac{dE_y(x)}{dx} \quad \text{(S2)}$$

The boundary conditions:

$$E_y^{\,u}(x)-E_y^{\,l}(x)=\begin{cases} 0 & x=\pm(d/2+t) \\ 0 & x=\pm d/2 \end{cases} \quad \text{(S3)}$$

$$H_z^{\,u}(x)-H_z^{\,l}(x)=\begin{cases} 0 & x=\pm(d/2+t) \\ -\sigma_g(\omega)E_y^{\,u}(x) & x=\pm d/2 \end{cases} \quad \text{(S4)}$$

where superscript *u* and *l* indicate the upper surface and the lower surface respectively. From equation (6) and (7), by taking the limit of the thickness $t\rightarrow0$, we get the dispersion relation of TE GSPs eigen modes of the coupled graphene waveguides, "$\pm$" corresponding to anti-symmetric mode and symmetric mode[4]:

$$\frac{\gamma_1}{(1\pm e^{-d\cdot\gamma_1})}=\frac{j\mu_0\omega}{2}\sigma_g(\omega) \quad \text{(S5)}$$

If we take the limit of the waveguide gap $d\rightarrow+\infty$ in equation (8), this means that there is no coupling between two graphene sheets, then formula (8) will be:

$$\beta=k_0\sqrt{\varepsilon_{1,r}-(\frac{\sigma_g(\omega)\eta_0}{2})^2} \quad \text{(S6)}$$

This is exactly the TE mode GSPs propagation constant of single-layer graphene sheet[5], where $\eta_0=\sqrt{\mu_0/\varepsilon_0}$ is the wave impedance in free space.

Similarly, we consider the TM mode GSPs:

$$E_x(x)=\begin{cases} Ae^{-\gamma_1(x-t-d/2)} & t+d/2<x<+\infty \\ Be^{\gamma_2(x-t-d/2)}+Ce^{-\gamma_2(x-d/2)} & d/2<x<t+d/2 \\ De^{\gamma_1(x-d/2)}+Ee^{-\gamma_1(x+d/2)} & -d/2<x<d/2 \\ Fe^{\gamma_2(x+d/2)}+Ge^{-\gamma_2(x+t+d/2)} & -t-d/2<x<-d/2 \\ He^{\gamma_1(x+t+d/2)} & -\infty<x<-t-d/2 \end{cases} \quad \text{(S7)}$$

$$H_y(x)=\frac{\omega\varepsilon_0\varepsilon_r}{\beta}E_x(x)\,,\,E_z(x)=\frac{j}{\beta}\frac{dE_x(x)}{dx} \quad \text{(S8)}$$

The boundary conditions:

$$E_z^{\,u}(x)-E_z^{\,l}(x)=\begin{cases} 0 & x=\pm(d/2+t) \\ 0 & x=\pm d/2 \end{cases} \quad \text{(S9)}$$

$$H_y^{\,u}(x)-H_y^{\,l}(x)=\begin{cases} 0 & x=\pm(d/2+t) \\ \sigma_g(\omega)E_z^{\,u}(x) & x=\pm d/2 \end{cases} \quad \text{(S10)}$$

From equation (12)、(13) and take the limit of the graphene thickness $t\to 0$, we get the dispersion relation of TM polarized GSP eigenmodes[1]:

$$\gamma_1(1\pm e^{-d\cdot\gamma_1})=\frac{2j\varepsilon_0\varepsilon_{1,r}\omega}{\sigma_g(\omega)} \tag{S11}$$

Taking the limit of the waveguide gap $d\to+\infty$ in equation (14), we get the TM surface wave propagation constant of single-layer graphene sheet[5]:

$$\beta=k_0\sqrt{\varepsilon_{1,r}-(\frac{2\varepsilon_{1,r}}{\eta_0\sigma_g(\omega)})^2} \tag{S12}$$

2. The van der Waals force (VWF) and the optical force

We compare the optical gradient force with the VWF to make sure that graphene will not be exfoliated from the adsorbed object. According to the intermolecular forces theory[6], when the distance between molecules is small, the energy of interaction between two molecules in a vacuum is $w(r)=B/r^{12}-C/r^6$, where B and C are the dispersion force coefficients and r is the distance between molecules. The first item in the right side of $w(r)$ represents strong repulsive force that determines how close two atoms or molecules can ultimately approach each other and the second item represents VWF. The VWF is calculated by $F_{VWF}=-\frac{dw(r)}{dr}\propto r^{-7}$. As F_{VWF} decays very quickly with increasing of r. Therefore, for convenient calculations, we derive the VWF between a monolayer graphene nanoribbon and an infinite-width Si slab to estimate the VWF between the graphene nanoribbon and the adhered free-standing Si waveguide. Finally we get the VWF: $F_{VWF}=\frac{-Aw\Delta}{2\pi}\left(\frac{1}{D_0^4}-\frac{1}{(D_{0+h})^4}\right)$, where Δ is the thickness of single-layer graphene, D_0 is the equilibrium spacing between the upper graphene nanoribbon and the adsorbed Si waveguide. The minimum $w(r)$ occurs at D_0 .A is the Hamaker constant. Taking a typical value of $A=\pi^2C\rho_1\rho_2=10^{-19}$ J, Δ=0.34 nm, D_0=0.5 nm, w=100 nm in our device, then the F_{VWF} is 8.65N/m. When optical power is 2.5 mW, the largest optical force between the upper graphene nanoribbon and substrate in our analysis is $7.32\cdot 10^{-3}$ N/m, which is much smaller than the VWF. In fact the upper graphene nanoribbon is double-clamped too, it not just adhere on the waveguide. Once the nanoribbon deflects slightly, there will be a tangential force along the nanoribbon. Owing to these two reasons, the graphene will not be exfoliated from the Si waveguide by optical force.

3. The Casimir forces, electrostatic force and the optical force

For dynamic tuning of chemical potential, electrostatic doping is generally used. In such case, electrostatic force should also be discussed. Here we calculate the electrostatic force by COMSOL. As two graphene sheets in our configuration have the same chemical potential, i.e. the same gate voltage. So the electrostatic force between them is repulsive force. As is show in Fig.S2 (a) the red arrows are the electrostatic field. We set μ_c=1 eV and corresponding to the V_g=8.9 V. The largest electrostatic force is 0.42pN/μm. So the electrostatic force can also be ignored.

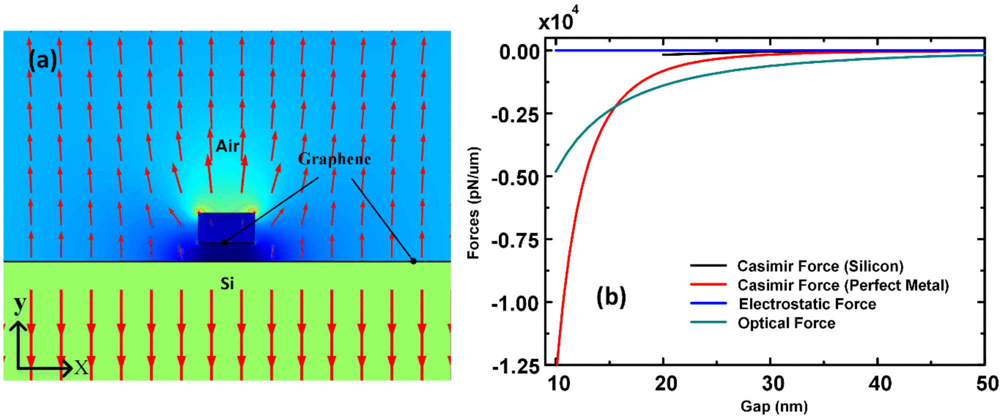

Fig.S2. The comparision of the Casimir forces, the electrostatic force and the optical force. (a) The electrostatic field distribution when gate voltage is applied. (b) The electrostatic force and the optical force in our structure and the Casimir forces.

Casimir forces are arising from the quantum vacuum fluctuations of the electromagnetic field at all frequencies[7,8]. According to the Lifshitz theory, the idealized Casimir force for perfectly conducting plates when temperature T=0 have the following forms[9]:

$$F_{c-idealized} = -\frac{\pi^2 \hbar c}{240}\frac{A}{d^4} \tag{S13}$$

where A is the area of plates, d is the gap between them. Usually T>0 corrections are negligible[10].

But due to the imperfect reflection, finite conductivity, the surface roughness and the finite thickness of practical structures, the actual Casimir force is therefore given by:

$$F_c = \eta(\mathrm{d}) \cdot F_{c-idealized} \tag{S14}$$

where η(d) is a finite correction factor and the actual force is therefore reduced by at least one order of magnitude[11]. As is shown in the Fig.1(b), when the gap is larger than 15 nm, the optical force in our configuration is larger than the idealized Casimir force between perfectly conducting plates. If fact, when the gap is 20 nm, the Casimir force between silicon plates is only about 165-pN/μm [8], which is much smaller than the optical force of our configuration. As the existence of graphene, our structure is relatively complicated, but the Casimir force is still smaller than that between perfectly conducting plates and may be larger than that between silicon plates. So we set the gap of our structure to be 30 nm and we believe that as long as the deflection is smaller than 15 nm, the Casimir force can be ignored.

References

1. Wang, B., Zhang, X., Yuan, X. & Teng, J. Optical coupling of surface plasmons between graphene sheets. *Appl. Phys. Lett.* **100**, 131111 (2012).
2. Lin, I.-T. & Liu, J.-M. Coupled surface plasmon modes of graphene in close proximity to a plasma layer. *Appl. Phys. Lett.* **103**, 201104 (2013).
3. Li, X. Y. H. a. R. Comparison of Graphene-Based Transverse Magnetic and electric surface plasmon modes. *IEEE J. Sel. Top. Quant* **21**, 62-67 (2014).
4. Povinelli, M. L. *et al.* Evanescent-wave bonding between optical waveguides. *Opt. Lett.* **30**, 3042-3044 (2005).
5. Hanson, G. W. Dyadic Green's functions and guided surface waves for a surface conductivity model of graphene. *J. Appl. Phys.* **103**, 064302 (2008).

6. Israelachvili, J. N. *Intermolecular and surface forces*. third edition edn, (Academic press, 2011).
7. Rodriguez, A. W., McCauley, A. P., Joannopoulos, J. D. & Johnson, S. G. Casimir forces in the time domain: Theory. *Phys. Rev. A* **80**, 012115 (2009).
8. McCauley, A. P., Rodriguez, A. W., Joannopoulos, J. D. & Johnson, S. G. Casimir forces in the time domain: Applications. *Phys. Rev. A* **81**, 012119 (2010).
9. Pan, K. *et al.* Calculation of nonzero-temperature Casimir forces in the time domain. *Phys. Rev. A* **83**, 040503 (2011).
10. Genet, C., Lambrecht, A. & Reynaud, S. The Casimir effect in the nanoworld. *Eur Phys J-Spec Top* **160**, 183-193 (2008).
11. Gusso, A. & Delben, G. J. Dispersion force for materials relevant for micro-and nanodevices fabrication. *J. Phys.D: Appl. Phys* **41**, 175405 (2008).
